# Small and dense LDL in familial combined hyperlipidemia and N291S polymorphism of the lipoprotein lipase gene

**DOI:** 10.1186/1476-511X-8-12

**Published:** 2009-03-31

**Authors:** Antonio López-Ruiz, María M Jarabo, María L Martínez-Triguero, Maria Morales-Suárez-Varela, Eva Solá, Celia Bañuls, Marta Casado, Antonio Hernández-Mijares

**Affiliations:** 1 Service of Endocrinology, University Hospital Dr Peset, Valencia, Spain; 2 Service of Clinical Analysis, University Hospital La Fe, Valencia, Spain; 3 CIBER CB/06/02/0045 research group, CIBER Actions in Epidemiology and Public Health, Valencia, Spain; 4 Medicine Department, University of Valencia, Valencia, Spain; 5 Institute of Biomedicina of Valencia (CSIC), CIBERehd, Valencia, Spain

## Abstract

There is a predominance of small and dense LDL cholesterol particles in familial combined hyperlipidemia (FCH). The lipoprotein lipase gene could exert an influence in these circumstances.

To study the relationship of pattern B LDL and lipids with N291S polymorphism of lipoprotein lipase (LPL) in FCH patients.

Lipid profile, apolipoproteins, diameter of LDL and N291S polymorphism were determined in 93 patients with FCH and 286 individuals from the general population.

FCH patients with N291S polymorphism showed a lower mean diameter of LDL. FCH patients with pattern B LDL showed higher concentrations of triglycerides, VLDLc, non-HDLc and apo B100 and lower levels of HDLc than those with pattern A. Of FCH patients with polymorphism 87.5% presented pattern B and 12.5% pattern A, while patients without polymorphism presented pattern A in 69.2% cases and pattern B in 30.8% cases, with differences being statistically significant (p < 0.004). The prevalence of this mutation in our FCH patients was 9.7%.

The prevalence of N291S mutation in our FCH patients was similar to the 9.3% described in Dutch FCHL patients but clearly higher than the 2–5% described for other Caucasian populations. No polymorphism was found in our general population sample. FCH patients with phenotype B of LDL possessed an atherogenic lipid profile. The relationship between small and dense LDL and the presence of the N291S mutation may identify patients with high cardiovascular risk.

## Background

A common form of dyslipemia associated with insulin resistance (obesity, diabetes mellitus, familial combined hyperlipidemia) is constituted by the presence of the atherogenic lipoprotein phenotype, characterized by elevated levels of triglycerides, small LDL particles and reduced HDL cholesterol [1]. This lipid triad frequently occurs in patients with premature coronary disease [2]. Small and dense LDL particles are formed largely as a response to high levels of triglycerides, and increase the risk of coronary heart disease [3]. LDL particles show a bimodal distribution in peak size and can be separated into two phenotypes; pattern A, in which larger, more buoyant LDL predominate, and pattern B, in which smaller, more dense LDL predominate. The National Cholesterol Education Program (ATP III) considers small and dense LDL as a lipid risk factor.

Familial Combined Hyperlipidemia (FCH; MIM 144250) is the most frequent genetic hyperlipemia. It is characterized by a tendency towards the appearance of coronary disease before 60 years of age [4]. The prevalence of this disease is 1–2% in the general population [5] and 10–20% among patients with premature coronary disease [6]. FCH presents a complex phenotype that is not completely understood, and which can vary between the patient and affected relatives [7]. Therefore, it is difficult to diagnose. It is also characterized by the presence of the atherogenic lipoprotein phenotype and an increase in apolipoprotein B100 [8].

Several genes have been studied in relation with FCH, which is a polygenic disease. One of the genes involved in FCH is lipoprotein lipase (LPL, E.C. 3.1.1.34) [9], a glycoproteic enzyme that plays a key role in the catabolism of lipoproteins rich in triglycerides, such as chylomicrons, and very low density lipoproteins (VLDL). LPL action takes place in endothelial cells surrounding the lumen of capillary vessels, where it adheres to heparan sulphate proteoglycans [10].

More than 80 structural mutations have been detected within the LPL gene. The frequency of these mutations in the general population is estimated to be at least 1/500. These heterozygous mutations are the main cause of some lipid disorders. One of these mutations is N291S localized in exon 6 of LPL [11-13].

The aim of our study was to analyze the relationship of the atherogenic lipoprotein profile, especially the predominance of small and dense LDL, with the prevalence of N291S LPL polymorphism in Spanish patients with FCH from the Community of Valencia with respect to with the general population.

## Materials and methods

Our study sample consisted of 33 families with Familial Combined Hyperlipidemia (FCH), with a mean of 4.4 individuals per family, all of them living in the Autonomous Community of Valencia (SE of Spain). From a total of 146 subjects, 93 were diagnosed with FCH according to the criteria of Bredie SJ et al. [14]. Total cholesterol and triglycerides levels superior to the 90^th ^percentile (adjusted to age and gender) and apo B100 values superior to 1.2 g/L represented a diagnosis of FCH in 43 men and 50 women, aged from 15 to 75 years. In order to assess polymorphism in the general population, 286 genetically independent healthy controls of similar gender and age were randomly selected.

Venous blood was collected for biochemical measurements. Total cholesterol (TC) and triglycerides (TG) were measured through enzymatic methods and high density lipoproteins cholesterol (HDLc) using a direct method in a Beckman LX20 auto analyzer. The intraserial variation coefficient was < 3.5% for all these determinations. When TG values were under 400 mg/dL, LDL cholesterol concentration was calculated using Friedewald's formule [15]. Non-HDL cholesterol was obtained from the difference between total cholesterol and HDL cholesterol. Apolipoproteins AI and B100 were determined by immunonephelometry in a Dade Behring BNII (intra-assay variation coefficient < 5.5%).

LDL B phenotype was measured by polyacrylamide gradient gel electrophoresis (2–16%) using the methodology described by Nichols et al. [16]. The assignment of LDL subclass phenotypes was based on particle diameter: pattern A, mean diameter > 25.5 nm, and pattern B, predominance of small and dense particles with a mean diameter ≤ 25.5 nm [17].

Approval was obtained from the relevant institutional review board, and all participants gave their informed consent.

### DNA analysis for RFLP detection in LPL

DNA was extracted from tubes of 15 mL of blood with EDTA-K_3_, according to the method of Miller et al. [18], and was conserved at 4°C until analysis was performed.

N291S was identified after PCR amplification of a 238 bp fragment using the following oligonucleotides: forward 5' GCCGAGATACAATCTTGGTG 3' and reverse 5' CTGCTTCTTTTGGCTCTGACTGT 3'. Such a polymorphism adds a restriction site for the Ava II enzyme. PCR reaction was performed in 25 μl total volume containing 0.3 mM of each primer, 0.2 mM of each dNTP, 1× Taq buffer, 2 mM MgCl_2_, 100 ng of genomic DNA and 2.5 U Taq polymerase (Netzyme) in a Master-Cycler thermocycler (Eppendorf). PCR was performed by means of a touchdown protocol [19] according to the following conditions: an initial denaturation step of 10 minutes at 94°C was followed by 4 cycles of 30 seconds at 94°C, 30 seconds at 60°C and 45 seconds at 72°C. This was followed by 20 cycles of 30 seconds at 94°C, 30 seconds at 60°C minus 0.5°C in each cycle and 45 seconds at 72°C, and then 10 cycles of 30 seconds at 94°C, 30 seconds at 50°C and 45 seconds at 72°C. The final extension consisted of 10 minutes at 72°C. The PCR product was digested for 4 hours at 37°C with Ava II and then analyzed with an 8% polyacrylamide gel. If the polymorphism was present, two fragments of 215 and 23 bp (HT+) appeared; if not, the site was destructed in the normal chain, which remained non-digested (238 bp: WT).

### Study design

The study was conducted based on a case-control design. Ninety-three FCHL patients were recruited and matched according to sex and age with a control group of 286 subjects from the general population.

### Statistics

Between-groups comparison was performed using an ANOVA followed by a parametric t-test. Due to skewed distribution of TG, TG analyses were performed on logarithmically transformed values. Statistical analyses were carried out using the Microsoft Excel Data Analysis Package (Microsoft, Inc.).

The Mann-Whitney nonparametric test was used to compare lipid concentrations in FCH patients with pattern A and those with pattern B. To test differences in triglycerides levels, values were logarithmically transformed prior to statistical analysis.

χ^2 ^was performed in order to determine whether populations were in Hardy-Weinberg equilibrium and in order to compare different phenotypes. Significance was set at p < 0.05.

Statistic analyses were carried out using the SSPS-15.0 program for Windows (SPSS Inc., Chicago, IL).

## Results

A total of 93 patients, all diagnosed with FCH, were studied: 43 men (46.3%) and 50 women (53.7%), aged between 15 and 75. 286 subjects from the general population were also studied. Lipid parameters are expressed in Table 1. Significant differences were observed between the two groups in all the variables studied. FCH patients showed higher levels of total cholesterol, triglycerides, LDLc, non-HDLc and apo B100, and lower levels of HDLc, apo A and a smaller LDL diameter.

**Table 1:** Lipid concentrations in cases (FCH patients) and controls.

	FCH Patients	Controls	P value*
Total cholesterol	238.67 ± 50.10	185.13 ± 28.30	0.001
Triglycerides	204.71 ± 192.16	88.27 ± 42.45	0.001
LDLc	156.85 ± 39.97	113.50 ± 25.20	0.001
HDLc	47.77 ± 17.18	53.93 ± 14.48	0.001
VLDLc	40.28 ± 36.52	17.65 ± 8.49	0.001
Non-HDLc	192.94 ± 48.68	131.50 ± 29.09	0.001
Apo AI	133.55 ± 32.52	152.79 ± 29.03	0.001
Apo B100	125.80 ± 28.11	95.25 ± 21.15	0.001
LDL diameter	25.45 ± 0.78	26.14 ± 0.74	0.001

* ANOVA Test

Values are expressed as mean ± standard deviation. Units are mg/dL in all parameters except LDL diameter (nm).

When non-mutated wild-type (WT) and heterozygote (HT) patients were compared, a smaller average diameter of LDL particles was observed among the latter, with the difference being statistically significant. Table 2 shows the lipid values of pattern A patients compared to those of pattern B patients. FCH patients with pattern B LDL displayed higher concentrations of triglycerides, VLDLc, non-HDLc and apo B100 and lower levels of HDLc than FCH patients with pattern A.

**Table 2:** Lipid concentrations in FCH patients with pattern A and pattern B

	Pattern A (n = 26)	Pattern B (n = 27)	P-value*
Total cholesterol	227.88 ± 41.86	238.03 ± 50.86	0.450
Triglycerides	127.80 ± 65.77	344.55 ± 234.40	0.001
HDLc	55.00 ± 18.54	35.59 ± 7.94	0.001
LDLc	147.50 ± 34.64	149.06 ± 53.11	0.569
VLDLc	25.53 ± 13.11	65.34 ± 43.71	0.001
Non-HDLc	172.88 ± 32.40	202.44 ± 50.71	0.022
Apo AI	144.00 ± 39.87	121.33 ± 20.49	0.029
Apo B100	118.13 ± 22.22	133.69 ± 30.21	0.042
ApoB/ApoA	0.90 ± 0.29	1.50 ± 0.35	0.022
Diameter	26.06 ± 0.57	24.86 ± 0.42	0.001

* Mann-Whitney test

Values expressed as mean ± Standard Deviation. Units are mg/dL in all parameters

Patients with polymorphism presented pattern B in 87.5% of cases and pattern A in 12.5% of cases, while patients without polymorphism presented pattern A in 69.2% of cases and pattern B in 30.8% of cases. These differences were statistically significant (p < 0.004).

N291S polymorphism of LPL occurred in 9.7% of FCH patients, while no individual in the study sample from the general population presented this mutation. Both samples were in Hardy-Weinberg equilibrium, as the patients showed a χ^2 ^= 0.216 and 2p = 0.6423 and the general population a χ^2 ^= 0 and 2p = 1.

## Discussion

FCH is a disease with a complex phenotype that is not completely understood, and remains difficult to diagnose. This dyslipemic syndrome is associated with disturbances in all lipoprotein fractions. The key underlying abnormality of this condition is elevation of VLDL due to overproduction or defective catabolism [20]. Small and dense LDL is the final expression of this catabolism. These particles are more easily oxidized and display a higher affinity for the extracellular matrix and a higher degree of retention in arterial wall than their larger, normal-sized counterparts [21]. Small and dense LDL has been accepted as an emergent cardiovascular risk factor by the National Cholesterol Education Programme (Adult Treatment Panel III) [22], as many studies have demonstrated that the predominance of these particles is associated with an increase in cardiovascular risk more than classic lipid variables [23,24]. FCH patients with phenotype B of LDL show a clearly atherogenic lipid profile. There is evidence that hypolipidemic treatment can modify the distribution of LDL subclasses [25]. Very high risk patients, including those with this atherogenic lipoprotein profile, may benefit from more potent lipid-lowering therapies [26].

It has been suggested that FCH is a heterogeneous and oligogenic disease [27]. One of the genes likely to exert an influence in this disease is the lipoprotein lipase gene. Many codon polymorphisms of the LPL gene have been described, some of them associated with alterations of the metabolism of triglyceride-rich lipoproteins [28]. One of the most frequent polymorphisms is located in exon 6 (rs268, c. 1128 A>G; N291S) [29]. The prevalence of this mutation in our FCH patients was 9.7%, which is similar to the 9.3% described for Dutch FCH patients [5] but considerably higher than the 2–5% described for other Caucasian populations [30]. No polymorphism was detected in the sample from the general population, which reflects findings in other populations [5] in which no carriers were found, suggesting that the N291S mutation is more frequent among FCH patients. The mutation is likely to affect postprandial lipemia by altering its enzymatic activity [31], resulting in an increase of VLDL particles and high concentrations of apo B100 and leading to the appearance of a greater number of small and dense LDL particles as the final and most important expression of the catabolism of triglyceride-rich lipoprotein particles [32]. In this way, the FCH phenotype of patients with the polymorphism is negatively affected, with altered postprandial responses to fat-rich meals being described, even in patients that were initially normolipemic [33].

These previously published data coincide with our results; polymorphism-carrier patients have been reported to present a smaller LDL diameter, suggesting a clearly atherogenic alteration of the metabolism of triglyceride-rich particles [34]. In fact, in some studies regarding FCH, this polymorphism has been related to patients who had previously presented an ischemic cardiopathy [11].

In our study, patients with the N291S polymorphism showed a smaller average LDL diameter, corresponding with pattern B and constituting the final expression of the alteration of the metabolism of triglyceride-rich particles. This represents a higher cardiovascular risk among such patients. On the other hand, most patients without this polymorphism had pattern A, constituting a clear difference between both groups. We are aware of the limitations of this study in relation to the number of patients with the polymorphism. Unfortunately, its low prevalence among the general population makes it difficult to conduct a genetic study with a large number of patients that would permit us to draw more solid conclusions. That said, our data show important differences between the two patterns.

In conclusion, FCH patients with pattern B of LDL show a clearly atherogenic lipid profile. The relationship between small and dense LDL and the presence of the allele encoding for N291S mutation of the LPL gene, present in a high percentage of our FCH patients, could disrupt the phenotype of the disease by altering the clearance of triglyceride-rich lipoproteins. Such phenotype expression is the basis for a more atherogenic profile. Therefore, based on our results in FCH patients in the Autonomous Community of Valencia, the cardiovascular risk of those with pattern B of LDL who carry this polymorphism could be considerably reduced through rigorous treatment of Hyperlipemia.

## Abbreviations

FCH: Familial combined hypermipidemia; LPL: lipoprotein lipase; WT: non-mutated wil-type; HT: Heterzygote.

## Competing interests

The authours declare that they have no competing interests.

## Authors' contributions

AH-M, AL-R and MM-T participated in the design of the study, MJ, AL-R and MM-T carried out the laboratory studies. CB and MM performed the statistical analysis and interpretation of data. MC and AL-R carried out the molecular genetic studies. ES and AH-M have been involved in the revision of the manuscript. All authors read and approved the final manuscript.

## Acknowledgements

MC was supported by grant SAF2006-06760 from CICYT, Spain and by a grant from Instituto de Salud Carlos III (Red de Centros FIS-RECAVA RD06/0014/0025).
